# The Achilles Heel of the Trojan Horse Model of HIV-1 *trans*-Infection

**DOI:** 10.1371/journal.ppat.1000051

**Published:** 2008-06-27

**Authors:** Marielle Cavrois, Jason Neidleman, Warner C. Greene

**Affiliations:** 1 Gladstone Institute of Virology and Immunology, San Francisco, California, United States of America; 2 Department of Medicine, University of California, San Francisco, California, United States of America; 3 Department of Microbiology and Immunology, University of California, San Francisco, California, United States of America; University of British Columbia, Canada

## Abstract

To ensure their survival, microbial pathogens have evolved diverse strategies to subvert host immune defenses. The human retrovirus HIV-1 has been proposed to hijack the natural endocytic function of dendritic cells (DCs) to infect interacting CD4 T cells in a process termed *trans*-infection. Although DCs can be directly infected by certain strains of HIV-1, productive infection of DCs is not required during *trans*-infection; instead, DCs capture and internalize infectious HIV-1 virions in vesicles for later transmission to CD4 T cells via vesicular exocytosis across the infectious synapse. This model of sequential endocytosis and exocytosis of intact HIV-1 virions has been dubbed the “Trojan horse” model of HIV-1 *trans*-infection. While this model gained rapid favor as a strong example of how a pathogen exploits the natural properties of its cellular host, our recent studies challenge this model by showing that the vast majority of virions transmitted *in trans* originate from the plasma membrane rather than from intracellular vesicles. This review traces the experimental lines of evidence that have contributed to what we view as the “rise and decline” of the Trojan horse model of HIV-1 *trans*-infection.

## Introduction

Dendritic cells (DCs) play a central role in initiating the adaptive immune response that counters pathogen infection. Immature DCs patrol the peripheral mucosal tissues, searching for unwanted intruders. Once a pathogen is sensed, captured, and internalized, DCs undergo a maturation process and migrate to the regional lymph nodes. Meanwhile, the internalized pathogens are processed into antigenic peptides, and co-stimulatory molecules are expressed on the cell surface, readying these professional antigen-presenting cells for effective T-cell stimulation [1]. To perform their key sentinel function, DCs express a repertoire of pathogen recognition receptors, including Toll-like receptors and C-type lectin receptors. Toll-like receptors relay pathogen alert signals to DCs through intracellular signaling pathways, culminating in both cellular maturation and cytokine production [2],[3]. C-type lectin receptors recognize specific carbohydrate structures on these pathogens and internalize them for degradation in lysosomal compartments, thus initiating the process of antigen presentation [4],[5].

Pathogens have evolved various means to escape the host immune response by subverting the function of DCs. HIV-1 excels in this capacity. Like many other microbial pathogens, HIV-1 interferes with Toll-like receptor signaling, impairing the secretion of antiviral and inflammatory cytokines needed for the development of an effective immune response [6],[7]. HIV-1 also likely uses DCs as a cellular ferry to reach one of its major targets, CD4 T cells, located deep within the mucosa or in lymph nodes. HIV-1 can also directly infect and replicate in Langerhans cells (LCs) and other myeloid DCs [8], although this infective process is much less robust than that in CD4 T cells and seems to require higher viral input [9]. Nevertheless, new virions budding from infected DCs can help provide an “infectious beachhead” for subsequent spread to CD4 T cells. In addition to replicating at low levels in DCs, HIV-1 has been proposed to exploit these cells for a novel form of viral spread involving the initial capture and internalization of intact virions, followed later by the transfer of virions across synapses formed by CD4 T cells scanning the DCs for cognate antigen. This mechanism of viral spread is termed *trans*-infection.

Although direct evidence of *trans*-infection in vivo is lacking, numerous in vitro observations suggest that such capture and transfer of virions to permissive cells is advantageous for the virus, in particular when quantities of infectious particles are limiting. In early studies of ex vivo tissue explants, most HIV-1 replication was observed within DC–T-cell conjugates [10],[11],[12]. DCs added to peripheral blood mononuclear cells also greatly enhance HIV-1 replication. In these cocultures, most virions originate from T cells, suggesting that DCs contribute in an indirect manner to overall viral production [13].

The mechanism underlying this DC-dependent enhancement of HIV infection remained unknown until the discovery of DC-specific intercellular adhesion molecule-grabbing non-integrin (DC-SIGN) [14]. DC-SIGN is a C-type lectin receptor expressed by mucosal DCs and DCs derived in vitro from monocytes or CD34 stem cells [14],[15] and a subset of macrophages [16],[17]. DC-SIGN binds with high affinity to the HIV-1 envelope protein gp120 but, unlike CD4, does not trigger viral fusion. Instead, this interaction promotes efficient virion capture [18],[19]. These captured virions are subsequently transmitted *in trans* to interacting CD4 T cells [18]. Thus, DCs facilitate productive infection of CD4 T cells while not serving as hosts for viral replication. Further studies indicated that *trans*-infection involved internalization of virions into an endocytic compartment [20],[21]. Virion-containing vesicles were later exported to the cellular synapses formed between interacting CD4 T cells and DCs [21]. These studies gave rise to the widely accepted concept of the “Trojan horse” model of *trans*-infection in which exocytosis of virion-laden vesicles into the synapse promoted highly efficient infection of CD4 T cells.

## Diversity of DC Receptors Involved in *trans*-Infection

DC-SIGN, the most-studied C-type lectin receptor that captures HIV-1 virions, is a calcium-dependent lectin that binds the HIV envelope with an affinity similar to that of CD4 [19]. The C-terminal domain of DC-SIGN interacts with unknown carbohydrate structures on gp120 [4],[22],[23],[24]. Expression of DC-SIGN in the lymphoblastoid cell line Raji (Raji-DC-SIGN), originally mistaken for the monocyte-type line THP-1, is sufficient to promote *trans*-infection of T cells [18],[25]. However, whether DC-SIGN is important in vivo remains controversial. Some reports indicate that *trans*-infection of T cells by DCs derived in vitro from monocytes (MDDCs) involves DC-SIGN [9],[18],[20],[26],[27], while other studies suggest the involvement of alternative C-type lectin receptors [16],[28],[29],[30]. DC-SIGN is expressed in vivo by some immature DCs located in mucosa within the lamina propria [31]. Nevertheless, further studies are needed to sort out the precise role of DC-SIGN versus other receptors in the capture of HIV-1 virions by these DCs.

Other C-type lectin receptors, such as langerin and mannose receptors, are at least equally important for gp120 binding to epithelial DCs [32],[33],[34]. In freshly isolated skin LCs, langerin binds gp120. In dermal DCs, binding involves mannose receptors. Interestingly, when dermal DCs migrate out of skin explants, C-type lectin receptors are down-regulated, and gp120 binding occurs predominantly through CD4 [33]. Direct evidence of *trans*-infection mediated by mannose receptors in DCs is lacking, but in macrophages, these receptors appear to play an important role in the *trans*-infection of CD4 T cells [35]. Conversely, capture of HIV-1 by langerin expressed by LCs does not lead to CD4 T-cell infection *in trans* [9]. Instead, langerin-bound virions are rapidly cleared, especially with low viral input. Inhibition of HIV-1 binding to langerin with a newly developed anti-langerin antibody or with mannan, a soluble general inhibitor for C-type lectin receptors, markedly increases viral replication in cocultures of T cells and epithelial LCs. This phenotype sharply contrasts with *trans*-infection mediated by DC-SIGN on MDDCs, suggesting that langerin and DC-SIGN may, in fact, mediate opposite fates for HIV-1 virions. However, caution is warranted when comparing results from ex vivo LCs and in vitro derived DCs. LCs derived in vitro from CD34-expressing precursor cells, like MDDCs, mediate efficient *trans*-infection of T cells despite expression of langerin [36]. The use of higher viral inputs in these later experiments might have overwhelmed the apparent protective function of langerin. Interestingly, maturation of LCs with lipopolysaccharide and TNF-α, although only slightly reducing langerin expression, greatly increased the ability of these cells to support *trans*-infection [36], even at low viral inputs [37]. Further follow-up studies are required to better characterize the intriguing potential “clearing function” of langerin in immature LCs and to understand why this protective activity is diminished during LC maturation.

## Some HIV-1 Virions Are Rapidly Internalized by DCs, While Others Remain at the DC Surface

As part of their normal function, DCs internalize pathogens and process proteins from these organisms into small antigenic peptides for subsequent presentation to CD4 T cells on MHC-II receptors. Typically, immature DCs display high levels of endocytic capacity while mature DCs are characterized by efficient antigen processing and presentation. Early in vitro and ex vivo studies supported the notion that DCs internalize structurally intact HIV-1 virions into large vacuolar structures [38],[39],[40]. At least some of these structures correspond to internal vesicles based on lack of co-staining with cationized ferritin [41] or ruthenium red [30], a small membrane-nonpermeable dye that binds to carbohydrates at the plasma membrane [42],[43]. Surprisingly, mature MDDCs harbor many more intact HIV-1 virions than immature MDDCs [41]. Numerous intact virions are found in mature MDDCs within large vesicles adjacent to the nucleus. In immature DCs, only a few virion-laden vesicles are detected, usually at the periphery of the DC. Virion internalization involves clathrin-dependent endocytosis based on visualization of virions within both clathrin-coated pits and internalized clathrin-coated vesicles [41],[44]. However, some virions remain detectable at the plasma membrane, notably within deep folds of the plasma membrane [30],[44] or between dendrites [45]. Surface-bound virions are also readily observed on LCs emigrating from vaginal epithelia [46]. In LCs isolated from skin biopsies and subsequently loaded with HIV-1, virions colocalize with Langerin at the cell surface and in Birbeck granules [9]. The latter are LC-specific cytoplasmic organelles likely involved in antigen processing [47].

In mature MDDCs, the compartment harboring HIV-1 virions shares certain features with the late endosome or the multivesicular body (MVB), but other features differentiate these structures from classical late endosomes or lysosomes [44],[45],[48]. Specifically, this compartment contains the tetraspanin receptors CD81, CD82, and CD9 but contains little CD63 and no LAMP-1, EEA-1, or TGN46, markers for lysosomes, early endosomes, and trans-Golgi networks, respectively [44],[49]. Immature MDDCs lack these structures, suggesting that mature and immature MDDCs may exhibit different intracellular trafficking patterns for HIV-1 virions [45]. In this regard, immature DCs may reorganize their endocytic compartments upon interaction with HIV-1 virions [44], perhaps due to triggering of DC maturation via stimulation of Toll-like receptor 8 [7].

Upon interaction with T cells, HIV-1-loaded MDDCs redistribute virion-containing vesicles to the DC–T-cell junctions [21],[44],[48], and CD4, CXCR4, and CCR5 are similarly recruited to the T-cell face of the synapse [21]. Further evidence of HIV-1 virions localizing within the intracellular space between the contact zone of T cells and MDDCs [30] or emigrant LCs [46] is found in electron microscopic studies. The cell–cell contact zone that facilitates HIV-1 transmission by locally concentrating virions and viral receptors was termed the “infectious synapse” (for review, see [50],[51]). Together, these findings argued in favor of the Trojan horse model, in which HIV-1 virions tap into an endocytic-exocytic process of DCs to mediate *trans*-infection of CD4 T cells [21],[30],[44],[45],[52].

## HIV-1 Virions Transmitted *in trans* to CD4 T Cells Originate from the DC Surface

**Figure 1:**
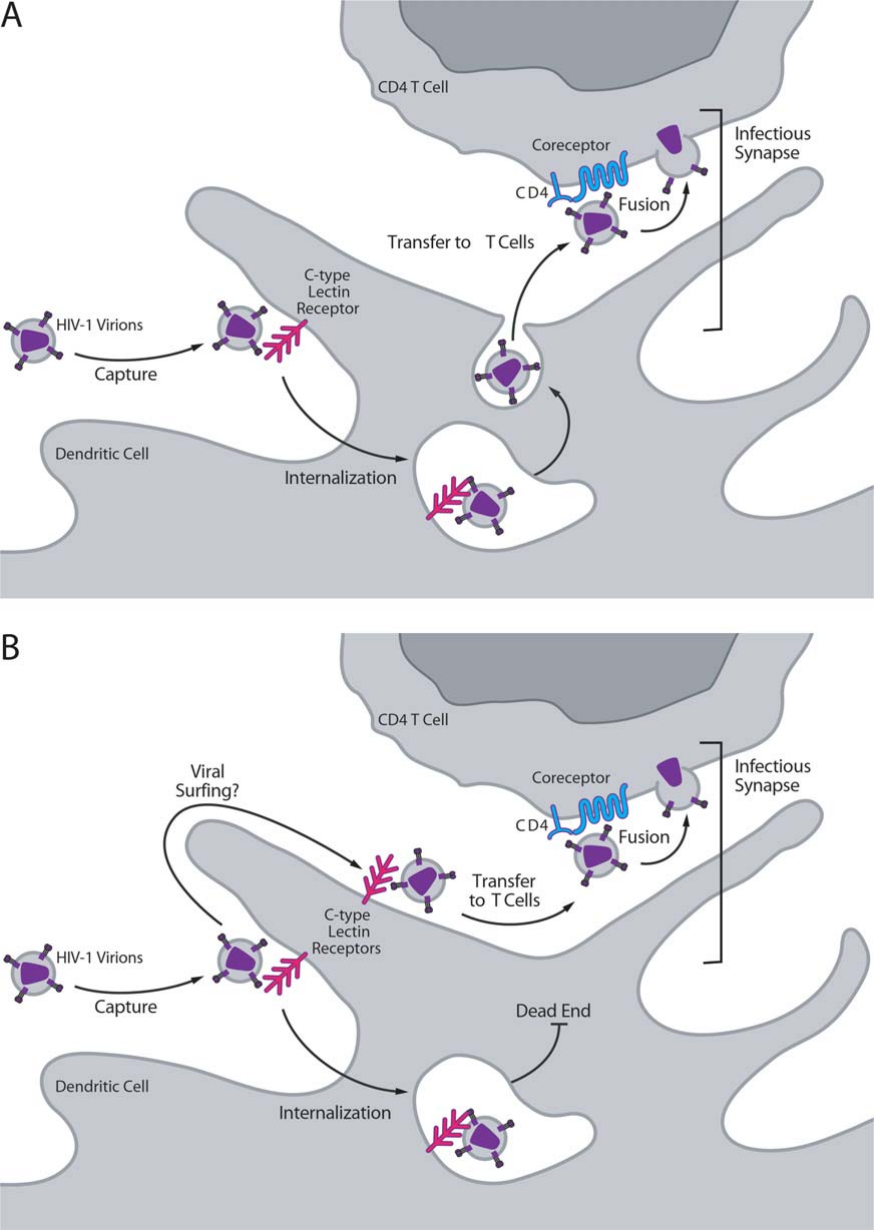
Models for *trans*-Infection of CD4 T cells by DCs. After capture by DCs, HIV-1 virions are either internalized or remain at the cell surface, possibly at the tips of dendrites or within extensively folded invaginations of the plasma membrane. A. In the prevailing Trojan horse model of HIV *trans*-infection, *trans*-infection is primarily mediated by internalized virions. B. In the new model, surface-bound HIV-1 virions are mainly responsible for *trans*-infection. Virions possibly surf the surface of the DCs on lipid rafts that collect at the infectious synapse to promote effective delivery to interacting CD4 T cells.

Seeking to better understand the events involved in HIV-1 *trans*-infection, we tested the effects of soluble CD4 (sCD4). This agent selectively neutralizes gp120 on surface-bound virions while not altering internalized virions. To our surprise, sCD4 completely inhibited HIV-1 *trans*-infection [36], raising the possibility that the surface-bound virions, rather than internalized virions, represent the major source of virus for *trans*-infection. To exclude possible unappreciated effects of sCD4, we also inactivated surface-bound virions with pronase. Again, *trans*-infection was abrogated by the selective incapacitation of surface-bound HIV-1 virions in both MDDCs and CD34-derived LCs. These findings implicating surface virions in *trans*-infection (Figure 1) are also supported by two prior studies employing cell-impermeable inhibitors [53],[54], which prevent viral fusion by disrupting the viral envelopes. In the first study, amphibian-derived peptides effectively inhibited *trans*-infection when applied to HIV-1-loaded MDDCs and thoroughly washed out before incubation with target cells [53]. Van Coppernolle et al. raised for the first time the possibility that virions transferred from DCs originate from the cell surface. However, in an attempt to reconcile these findings with the strongly prevailing Trojan horse model, the authors proposed an alternative explanation, in which HIV-1 virions were targeted by small quantities of residual peptide remaining after the wash procedure at a time when vesicle contents are released into the synaptic cleft. In our studies evaluating possible “carryover” effects of sCD4, we find that sCD4 only inactivates virions accessible at the time of sCD4 treatment and not virions loaded after the application of sCD4 and its removal by washing [36]. In the other study, a novel topical microbicide candidate called SAMMA [55], which appears to interrupt HIV-1 gp120/gp41 fusion, also potently inhibited *trans*-infection of T cells by MDDCs [54] without gaining entry into cells. Together, these findings are consistent with our conclusion that *trans*-infection is primarily mediated by surface-bound rather than internalized HIV virions.

The only situation in which a few internalized virions may find their way to the infectious synapse and infect interacting T cells *in trans* is in the context of specific antigen recognition by interacting CD4 T cells. Recognition of antigens loaded on MHC-II at the DC surface by the appropriate T-cell receptors triggers the rearrangement of the MVB, promoting additional MCH-II receptor translocation from the MVB to the DC surface [56]. Staphyloccal enterotoxin B mimics this sequence of events by cross-linking the variable region of the TCR β-chain of T cells and MHC-II molecules [57]. In this setting and this setting alone, we detected very low levels of transfer of internalized virions to interacting CD4 T cells. However, this transfer represents less than 1% of the total transfer events; 99% or more of virions mediating *trans*-infection continue to derive from the cell surface [36]. Nevertheless, it is intriguing to consider the possibility that this transfer might play a role in the preferential and rapid depletion of HIV-1-specific CD4 T cells that occurs in HIV infection [58].

## The Rise and Decline of the Trojan Horse Model for *trans*-Infection

The Trojan horse metaphor became popular with the report of Steinman et al., which showed that the potency of DCs in stimulating *trans*-infection of CD4+ T cells [10]. This metaphor emphasized that DCs, a trusted party in the immune system, could actually carry an infectious agent to CD4+ T cells, leading to their destruction. The mechanism was refined later with the discovery of DC-SIGN as an HIV-1 gp120 binding protein [18]. The analogy to the Trojan horse became even more striking when Kwon et al. [20] suggested that HIV-1 virion internalization was required for *trans*-infection of T cells. The strongest support for this conclusion was the ability of both immature MDDCs and Raji-DC-SIGN cells loaded with R5-tropic HIV-1 virions to *trans*-infect T cells despite the inactivation of surface-bound virions with trypsin. While these experiments included appropriate controls indicating that the trypsin had efficiently inactivated surface-bound virions, the study did not exclude direct infection of immature MDDCs as the source of viral reporter expression. To take this analysis one step further, we performed similar experiments with reporter viruses encoding two distinct epifluorescent proteins, allowing clear distinction between signals deriving from DCs and T cells [36]. Again, we observed that inactivation of surface-bound HIV virions completely abrogated *trans*-infection by MDDCs. Direct infection of Raji-DC-SIGN cells could not readily explain the results obtained, as these cells are only weakly susceptible to HIV-1 infection [59]. However, two recent reports employing trypsin or pronase treatment of Raji-DC-SIGN cells suggest that *trans*-infection by these cells involves virions located at the cell surface [29],[30]. The initial study also concluded that Raji cells expressing DC-SIGN with a truncation of its cytoplasmic domain failed to *trans*-infect T cells. However, the large truncation in DC-SIGN might also have affected formation of the infectious synapse, which seems to require DC-SIGN or the effective migration of DC-SIGN-bound virions on the DC surface to the infectious synapse [27]. Of note, a subsequent study highlights how point mutations that compromise the internalization of DC-SIGN do not impair *trans*-infection [60].

Striking time-lapse microscopic images of internalized virions being delivered to interacting CD4 T cells via the infectious synapse added further momentum to the Trojan horse model [21]. However, because of the nature of these experiments, it was not possible to determine if the vesicles moving to the infectious synapse ferried infectious virions. The recruitment of these virions to the synapse was also observed after trypsin digestion of HIV-loaded DCs, although data on the completeness of surface virion inactivation were not presented.

In subsequent papers, proteolytic digestion remained the principal tool for eliminating surface virions as a source of virus-mediating *trans*-infection [29],[37],[61]. The effectiveness of these proteases varied greatly from study to study (Table 1). Numerous parameters influence protease effectiveness, including the type of protease used, the time and temperature of incubation, and the complexity of the cell membrane. Appropriate controls are essential to ensure that all surface-bound virions are in fact inactivated. Cleavage of DC-SIGN receptors at the surface of MDDCs has been used as one control to monitor protease effectiveness [30]. However, despite a decrease in surface expression of DC-SIGN, most surface-bound virions (87%) remained attached to mature MDDCs. Thus, DC-SIGN cleavage is not a reliable control for proteolytic inactivation of surface virions, particularly in mature MDDCs. Accordingly, *trans*-infection experiments employing this criterion for virion inactivation must be interpreted with great caution. Internal controls, as initially described by Kwon et al. [20], remain the best approach. These authors used two reporter viruses, one internalized and the second bound to the DC surface, to serve as an internal control for the completeness of inactivation of surface-bound virions. When we performed similar experiments with reporter viruses that permit the distinction of signal emanating from productively infected DCs and actual *trans*-infection of T cells, we did not observe any contribution of internalized virions in DCs to the *trans*-infection process under conditions in which surface virions were fully inactivated with pronase [36].

**Table 1:** Variable Effects of Protease Treatment on HIV *trans*-Infection.

Cells	Inhibition of *trans*-Infection (% of Untreated)	Treatment	Controls^a^	Reference
Raji-DC-SIGN	∼35%	Trypsin	Internal controls	[20]
	91%	Trypsin	Cleavage of DC-SIGN	[30]
	∼90%	Pronase	Cleavage of DC-SIGN	[29]
Immature MDDCs	∼45%–50%	Trypsin	Internal controls	[20]
	40%–50%	Trypsin	Cleavage of DC-SIGN	[30]
	48%–51%	Pronase	Cleavage of DC-SIGN	[30]
	99%	Pronase	Internal controls	[36]
Mature MDDCs	20%	Trypsin	Cleavage of DC-SIGN	[30]
	24%–35%	Pronase	Cleavage of DC-SIGN	[30]
	99%	Pronase	Internal controls	[36]
Immature CD34-derived LCs	∼85%	Trypsin	Not presented	[37]
Mature CD34-derived LCs	∼83%	Trypsin	Not presented	[37]
Cord blood–derived DCs	0%	Pronase	Cleavage of DC-SIGN	[29]

a Controls used to assess the effectiveness of proteolytic digestion in removing surface-bound virions.

A major drawback of protease treatment of the cell surface is its relative lack of specificity for bound HIV-1 virions. Key components of the infectious synapse could be digested, resulting in altered or delayed synapse formation. The use of more specific membrane-impermeable inhibitors, such as sCD4 and HIV-1-neutralizing antibodies, avoids these complications. Two studies with these agents have appeared [36],[62]. We reported that sCD4 treatment of MDDCs and CD34-derived LCs loaded with HIV virions fully abrogated *trans*-infection of T cells [36]. Ganesh et al. used neutralizing antibodies but found no effect on HIV *trans*-infection of activated T cells by mature MDDCs [62]. While sCD4 neutralization of surface-bound virions was controlled internally, the effectiveness of antibody neutralization of surface-bound virions was not tested. Rather, neutralization of cell-free virions was presented. At the highest concentration of antibody used, Ganesh et al. achieved only 70% neutralization. Even higher quantities of antibody might be required to neutralize MDDC-bound virions, compared to free virions. Indeed, our preliminary data support this notion. When we have tested higher concentrations of neutralizing antibodies that achieve full inactivation of surface-bound virions, we observe that HIV-1 *trans*-infection is completely abrogated (unpublished data).

Recently, a twist on the Trojan horse model emerged in a study suggesting that HIV-1 virions in immune complexes may recover their infectivity after capture by immature MDDCs and release of the bound antibodies within the acidified endocytic compartment [63]. However, no data were presented to clearly show that this recovery of infectivity was occurring in an internal acidified compartment rather than at the plasma membrane. More studies are warranted to examine *trans*-infection in the context of opsonized HIV-1 virions.

## Internalization Is Likely a Dead End for *trans*-Infection

The fact that virions are almost exclusively transmitted from the DC surface implies that virion internalization is chiefly a dead end for infectious virions. Several factors influencing the internalization of HIV-1 virions might affect their likelihood to *trans*-infect T cells. Such factors include the state of DC activation and maturation, the time elapsing between virion capture by DCs and contact with interacting T cells, and the nature of receptors that mediate binding of HIV-1 virions.

External molecules are taken up in DCs via multiple pathways, including phagocytosis, macropinocytosis, and receptor-mediated endocytosis via clathrin-coated pits and caveolae. DC differentiation and maturation are associated with a decline in endocytic activity, particularly macropinocytosis [1]. This decrease in internalization with maturation might leave more intact virions at the surface of mature DCs and could contribute to the enhanced ability of mature DCs to *trans*-infect T cells [21],[36],[37],[45],[64],[65],[66]. Increased interactions between mature DCs and T cells may also facilitate virion transfer [21],[64]. Finally, differences in the nature of the compartment harboring virions could play a role. Large vesicles filled with intact HIV-1 virions are observed in mature DCs [30],[41],[48] but not in immature cells [41]. Whether these virus-filled structures are in fact contiguous with the plasma membrane is unknown, but this is certainly possible, given the exceedingly complex 3-D architecture of the DC plasma membrane. In this regard, two recent studies of HIV-1 budding in macrophages indicate that virions accumulating in apparently intracellular vacuolar structures are, in fact, budding from an extensively folded region of the plasma membrane [43],[67]. These structures, which are present in infected and uninfected macrophages, seem to be stable and could correspond to specialized compartments of the plasma membrane [67]. In mature MDDCs, images of virus-filled structures in direct continuity with the DC surface [30],[41] were initially interpreted as areas of active virion internalization or as exocytic vacuoles releasing captured virions. Such structures might in fact correspond to deep invaginations of the plasma membrane. This possibility is bolstered by the fact that in mature MDDCs ∼80% of vacuoles harboring the virions remain accessible to small membrane-impermeable tracer proteins like horseradish peroxidase applied at 4°C [44]. Future studies will likely better define the nature of these virion collection depots and determine whether these virions in fact reside in internal vacuoles or instead represent complex invaginations of the plasma membrane.

The time elapsing between virion capture by DCs and contact with interacting T cells may also affect the efficiency of *trans*-infection. In initial studies, virions captured by Raji-DC-SIGN and MDDCs retained their infectivity for several days [18],[68],[69]. However, more recent studies suggest that virions remain active for *trans*-infection for only a few hours after capture [30],[36],[48],[59],[60]. Low levels of direct HIV-1 infection of Raji-DC-SIGN and immature MDDCs could provide an explanation for the early reports of persistent *trans*-infection [30],[48],[59]. Currently, virion inactivation/degradation after DC capture is believed to underlie the rapid decline in *trans*-infection [30],[37],[48],[69]. However, rapid internalization of HIV-1 virions might also contribute to this quick decline in *trans*-infection, as seen in macrophages [35]. Treatment of macrophages with endocytosis inhibitors, such as cytochalasin D and chlorpromazine, increases the longevity and transfer of captured virions. These agents have not yet been tested in the context of *trans*-infection of T cells by DCs. Interestingly, the use of inhibitors that impair intracellular trafficking and/or acidification of the endosomes appear to exert only minimal effects on *trans*-infection [30],[59], although conflicting results have been obtained with the vacuolar ATPase inhibitor concanamycin A [20],[59]. Surface molecules other than C-type lectins could also influence the efficiency of *trans*-infection [61]. For example, expression of CD4 receptors in Raji cells increases internalization of virions and reduces *trans*-infection. Conversely, expression of Nef in MDDCs and neutralization of CD4 by antibodies decrease available surface CD4 and are associated with increases in *trans*-infection. Collectively, these studies suggest that virion internalization negatively regulates *trans*-infection and are consistent with surface-bound virions forming the principal source of virions mediating *trans*-infection.

## Future Directions

The finding that *trans*-infection of T cells by DCs involves primarily surface-bound virions argues that future research should be refocused on how HIV-1 hijacks the plasma membrane rather than the intracellular trafficking pathway as suggested by the original Trojan horse model. Unraveling how these virions are recruited to the infectious synapse is crucial. The presence of C-type lectin receptors in lipid rafts [70] suggests that HIV-1 virions likely reach the infectious synapse by “surfing” the surface of the plasma membrane of DCs on lipid rafts. Further studies to characterize the domain(s) of DC plasma membrane that serves as a source of infectious virions could reveal some similarities with the compartments in which HIV-1 buds in macrophages. Since that internalization seems to be mostly a dead end for infectious virions, elucidating how HIV-1 manages to remain at the cell surface poised for transfer *in trans* will be important. Finally, our new model suggests that in vivo transmission of virions captured by DCs to T cells is likely to be far more sensitive to attachment inhibitors and neutralizing antibodies than previously anticipated. Only time will tell whether this fact can be therapeutically exploited.
